# ﻿*Pipermotuoense*, a new species of Piperaceae from Xizang, China

**DOI:** 10.3897/phytokeys.238.115494

**Published:** 2024-02-07

**Authors:** Fan Su, Xiao-Wei Qin, Rui Fan, Lin Yan, Xun-Zhi Ji, Chao-Yun Hao

**Affiliations:** 1 Spice and Beverage Research Institute, CATAS, Wanning 571533, Hainan, China Spice and Beverage Research Institute Wanning China; 2 Ministry of Agriculture Key Laboratory of Genetic Resources Utilization of Spice and Beverage Crops, Wanning 571533, Hainan, China Ministry of Agriculture Key Laboratory of Genetic Resources Utilization of Spice and Beverage Crops Wanning China; 3 Hainan Provincial Key Laboratory of Genetic Improvement and Quality Regulation for Tropical Spice and Beverage Crops, Wanning 571533, Hainan, China Hainan Provincial Key Laboratory of Genetic Improvement and Quality Regulation for Tropical Spice and Beverage Crops Wanning China

**Keywords:** Asia, Paleotropical flora, Piperales, Sino-Himalaya, taxonomy

## Abstract

*Pipermotuoense* X.W.Qin, F.Su & C.Y.Hao, a new species of Piperaceae from Xizang, China, is described and illustrated in this paper. The new species resembles *P.yinkiangense* and *P.anisotis*, but it can be readily distinguished from the compared species by several characteristics. Gonophyll leaves are chartaceous and the leaf secondary vein count is 7–9, with the outermost pair being very weak when there are nine veins. Additionally, the apical pair arises 2–4 cm above the base and the leaf base is asymmetrical, with bilateral petioles that cling and heal together. Pistillate floral bracts are sessile, with 3, 4 or 5 stigmas. The description of the new species includes photographs, detailed descriptions, notes on etymology, distribution and habitat, as well as comparisons with morphologically similar species.

## ﻿Introduction

*Piper* L. is the nominate genus of Piperaceae and one of the most diverse lineages amongst basal angiosperms (Tebbs 1993; Soltis et al. 1999). This genus was established, based on the species *P.nigrum* L. from India (Sen and Rengaian 2022). The genus is considered to comprise approximately 2,000 species, mainly distributed in the Tropics (Gentry 1982; Kubitzki et al. 1993; Marquis 2004; Quijano-Abril et al. 2014; Jaramillo et al. 2023; Suwanphakdee et al. 2023). Distinctive characteristics of *Piper* include swollen stem nodes and minute, usually unisexual flowers compacted together on a fleshy rachis in Asian *Piper* species. Its flowers lack perianth and consist only of the staminate and pistillate reproductive parts, which are subtended by 1–3 floral bracts. The number of stamens varies from 3–12 (Suwanphakdee and Chantaranothai 2014). The anther is distinguished by 2–4 thecae, with longitudinal or transverse dehiscence. Asian taxa of *Piper* have been studied in numerous publications and are currently estimated to consist of over 600 species (Wallich 1824–1849; Blume 1826; Hooker 1886; De Candolle 1910, 1912, 1923; Ridley 1924; Backer and Bakhuizen van den Brink 1963; Long 1984; Huber 1987; Gardner 2006; Suwanphakdee et al. 2006, 2008, 2011, 2012, 2014; Asmarayani 2018).

Up to the present, more than 60 species have been recorded in China, half of which are endemic (Gilbert and Xia 1999; Cheng et al. 1999; Gajurel et al. 2001; Hao et al. 2012, 2015, 2017, 2020; Yang et al. 2017; Su et al. 2022) . Some species are economically important. A typical example is *P.nigrum* L. (Linnaeus 1753), which is the source of black pepper, the world’s most widely used spice (Takooree et al. 2019). A few other species, such *P.betle* L., *P.cubeba* L.f., *P.longum* L., *P.magen* B.Q.Cheng ex C.L.Long & Jun Yang bis and *P.pedicellatum* C.DC., are used locally as condiments or medicine (Yang et al. 2017; Salehi et al. 2019) .

Through two field investigations over an interval of three years in Motuo County, Xizang, China, specimens of a dioecious plant were found and collected. Based on a detailed examination of the morphological characteristics of this plant and its possible relatives (Tseng 1979; Cheng et al. 1999; Gilbert and Xia 1999; Suwanphakdee and Chantaranothai 2011; Suwanphakdee and Chantaranothai 2014; Su et al. 2022; Hao et al. 2012, 2015, 2017, 2020; Junior and Guimaraes 2015; Mathew et al. 2016; Mukherjee 2016, 2018, 2020), we concluded that it did not match morphologically with any of the existing species. It exhibits trophophyll blades with an auriculate-cordate and asymmetrical base, with base bilaterally clung to the petiole and overlap together, leaf-blades abaxially densely villous, especially along the mid-vein and flowers with a single stamen. Due to these distinctive morphological features, we confirm that it is a new species, which we describe and illustrate here as *Pipermotuoense* X.W.Qin, F.Su & C.Y.Hao.

## ﻿Material and methods

Morphological studies of the new species were conducted, based on the type specimens deposited in the Herbarium IBSC and the living plants cultivated in the Spice and Beverage Research Institute, CATAS. All available specimens of *Piper* stored in the Herbaria of AU, BM, E, G, HITBC, IBK, IBSC, K, KUN, PE and WU were examined using online specimen images via the Chinese Virtual Herbarium (CVH, https://www.cvh.ac.cn/index.php) and JSTOR (https://plants.jstor.org). Measurements of morphological characters were taken from living plants and photographs were captured using a Nikon Z7 digital camera (Tokyo, Japan) and Dino-Lite digital microscope (Taiwan, China). Morphological comparison with closely-related species was made by consulting published literature.

## ﻿Taxonomy

### ﻿ Piper motuoense

Taxon classificationPlantaePiperalesPiperaceae

X.W.Qin, F.Su & C.Y.Hao sp. nov.

24A792C8-C689-5716-AE74-229B484DF470

urn:lsid:ipni.org:names:77335908-1

Figs 1 , 2

#### Diagnosis.

The new species is morphologically similar to *P.yinkiangense*, but can be easily distinguished from the latter in several aspects. The leaf-blades 12.5–18 × 3.5–6.5 cm, elliptic or ovate to lanceolate (vs. 11–14 × 6.5–8.5 cm, oblique-ovate), chartaceous (vs. membranous), abaxially sparsely villous along the mid-vein (vs. abaxially sparsely hispidulous), gonophyll leaves 7–9 secondary vein pairs, the outer pair arising 2–4 cm above base (vs. 8–9, outer pair arising 1–2 cm above base), base bilaterally clinging to the petiole and overlap together (vs. basal sinus 1–2 mm wide on side of longer and wider lobe, 4–5 mm wide on other side, bilaterally free for 2–3 mm), floral bracts sessile (vs. petiolate) and stigmas 3, 4 or 5 (vs. 4). *P.motuoense* also resembles *P.anisotis* in the shape of leaves and fruit, but differs from the latter in the leaves vein 7–9 (vs. 5–7), leave base bilateral clinging to petiole and overlap together (vs. bilateral free for 1–2 mm) (Table 1).

**Table 1.** Morphological comparison of key characteristics in *P.motuoense*, *P.yinkiangense* and *P.anisotis*.

Characters		*P.motuoense*	*P.yinkiangense*	*P.anisotis*
Stem		Densely villous when young, glabrous when mature	Densely villous	Densely short tomentose
Gonophyllleaves	petiole	2.5–3 cm long	Ca. 2 mm long	Ca. 3 mm long
	blade	12.5–18 × 3.5–6.5 cm, elliptic or ovate to lanceolate, chartaceous, abaxially densely villous, especially along the veins, adaxially sparsely villous along the mid-vein	11–14 × 6.5–8.5 cm, oblique–ovate, membranous, abaxially densely pubescent, usually along veins, adaxial sparsely hispidulous	7.5–13 × 2.5–5 cm, oblique-oblong, membranaceous, abaxially densely villous, especially along the veins, adaxially densely hispidulous
	base	Auriculate-cordate, bilaterally clinging to leaves petiole and overlap together	Obliquely auriculate-cordate, basal sinus 1–2 mm wide on side of longer and wider lobe, 4–5 mm wide on other side, bilateral difference to 2–3 mm	Unequal-sided and more cordate, basal sinus 1–2 mm wide on side of longer and wider lobe, 3–4 mm wide on other side, bilateral difference to 1–2 mm
	secondaryveins	7–9, when 9 veins, the outermost pair is very weak, apical pair arising 2–4 cm above base	8–9, apical pair arising 1–2 cm above base	5–7, apical pair arising 1.3–2.5 cm above base
Pistillate spikes	spikes	4–5 × 0.5–0.7 cm	3 × 0.4 cm	1.5–3 × 0.4–0.6 cm
	peduncles	2–3 cm long, slightly shorter than spike	2.5 cm long, equal to or longer than spike	2 cm long, equal to or longer than spike
	floral bracts	suborbicular, sessile	suborbicular, short-pedicellate	suborbicular, short-pedicellate
	stigmas	3–4–5, 0.8–1 mm long	4, ca. 1 mm or longer	4, ca. 1 mm or longer
Fruit		3–3.5 × 2.5–3 mm	Ca. 3 mm in diam.	Ca. 3 mm in diam.

**Figure 1.**
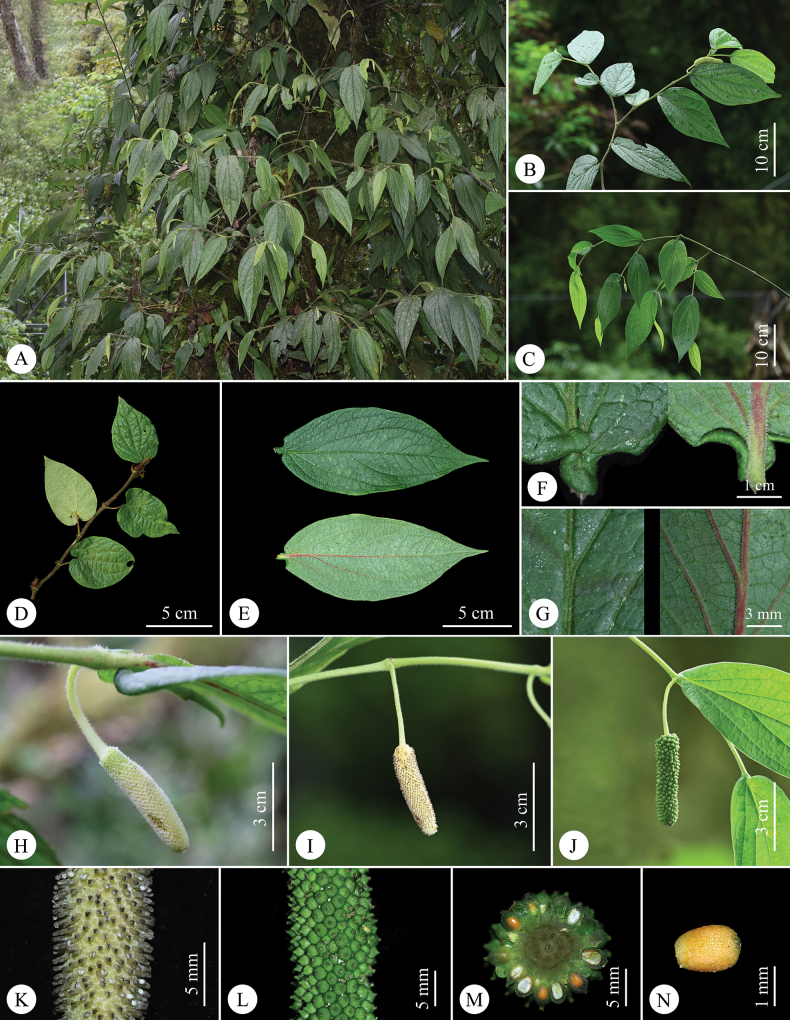
*Pipermotuoense* X.W.Qin, F.Su & C.Y.Hao, sp. nov. **A** habit **B** branch with infructescence **C** branch with staminate spike **D** branch with trophophyll leaf **E** adaxial and abaxial surface of gonophyll leaf **F** adaxial and abaxial surface of gonophyll leaf base **G** detail of the indument along the mid-vein of the gonophyll leaf adaxial and abaxial surface **H** pistillate spike **I** staminate spike **J** infructescence **K** close-up of portion of the staminate spike **L** close-up of portion of the infructescence **M** cross-section of infructescence **N** seed (side view). Photographs by Fan Su.

#### Type.

China. (Xizang): Linzhi, Motuo City, Beibeng, climbing on the taller trees in tropical rainforest, 29°10′48″N, 95°00′06″E, elevation ca. 490 m, 3 Oct. 2021, *Xiao-Wei Qin et al. 20211003, 20231016* (Holotype: IBSC0918558; Isotype: IBSC0918559, IBSC0918560, IBSC0918561).

**Figure 2.**
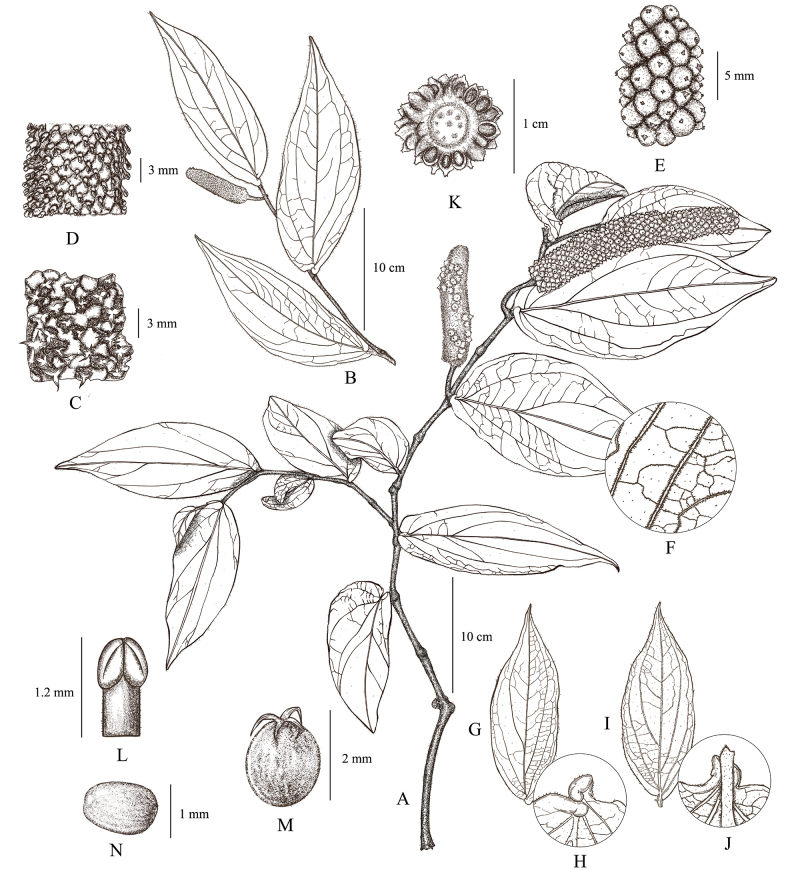
Line drawing of *Pipermotuoense* X.W. Qin, F. Su & C.Y. Hao **A** branch with infructescence **B** branch with staminate spike **C** magnified view of pistillate spike **D** magnified view of staminate spike **E** magnified view of infructescence **F** detail of the indument along the secondary nerves of the abaxial leaf surface **G** adaxial surface of gonophyll leaf **H** adaxial surface of gonophyll leaf base **I** abaxial surface of gonophyll leaf **J** abaxial surface of gonophyll leaf base **K** cross-section of infructescence **L** stamen **M** carpel **N** seed. Illustration by Fan Su, based on the holotype.

#### Description.

***Lianas*** over 5 m long, with climbing adventitious roots, dioecious, perennial, densely villous when young, becoming glabrous when mature. ***Roots*** dimorphic; basal roots terrestrial, feeding; adventitious roots produced along the aerial nodes, clasping the phorophyte. ***Stems*** climbing upwards, internodes terete, striated, with red stripes when they are young, swollen nodes. ***Leaves*** dimorphic, distichously-alternate, petiolate, blades chartaceous, glandular. ***Trophophyll leaves*** with petiole 2.5–3 cm long, cylinder-shaped in cross-section, red, pubescence; leaf-blades 7–9 × 5–6 cm, cordate to elliptic, adaxially dark green, glabrous, abaxially surface pale green, sparsely villous, especially along the veins, base usually cordate, symmetric, apex short-acuminate to long-acuminate; mid-vein red, 5–7 pairs, red, all basal, when 7, the outermost pair inconspicuous. ***Gonophyll leaves*** with petiole 0.3–0.4 cm long, cylinder-shaped in cross-section, pale green, densely pubescence; leaf-blades 12.5–18 × 3.5–6.5 cm, elliptic or ovate-lanceolate; 7–9 pairs of secondary veins; when 9 veins, the outermost pair is very weak; apical pair 2–4 cm above base, reaching leaf apex, alternate, others basal, reticulate veins prominent; adaxial surface dark green, glabrous or sparsely villous on the mid-vein, abaxial surface pale green, densely villous, especially on the veins; base auriculate-cordate, asymmetrical, bilateral clinging to leaves petiole and healing together, apex long acuminate. ***Inflorescence*** a pedunculate spike, leaf-opposed, pendulous; peduncle flexible, cylindrical; spadix cylindrical, the fertile rachis hairy, with densely compacted flowers; floral bracts sessile, imbricate, ca. 1 mm in diam., orbicular or suborbicular, piligerous, margin irregular, undulate. ***Staminate inflorescences*** 5.5–8 cm long, peduncles 3–4 cm long, villous; spike 2.5–4 × 0.5–0.7 cm, villous, pale green when young, pale yellow to white when mature; **stamens** 1, filaments 0.6–1 mm long, stout, hyaline, anthers 0.4–0.7 × 0.2–0.5 mm, 2-thecous, reniform, white before dehiscence, black after dehiscence, dehiscence lateral. ***Pistillate inflorescences*** 6–8 cm long, peduncles 2–3 cm long, piliferous; spike 4–5 × 0.5–0.7 cm, pale green when young, pale yellow to white when mature; **ovary** 1.6–2 × 1.4–1.8 mm, sessile, free from the neighbouring ones, obovoid, green, **style** 1–1.2 mm long, **stigmas** 3–5, 0.8–1 mm long, filiform, reflexed, cream-coloured at anthesis, becoming tan to light grey post-anthesis. ***Infructescence*** leaf-opposed, 5–7 × 1–1.2 cm; cylindrical; with densely compacted fruits; pendulous, cylindrical, piliferous. ***Drupes*** 3–3.5 × 2.5–3 mm, sessile, free from the neighbouring ones, subglobose, remaining attached to rachis at maturity, piligerous, persistent style 0.3–0.5 mm long, cylindrical; epicarp green, mesocarp pale green, translucent, endocarp dark yellow. ***Seeds*** 1–1.2 × 0.6–0.8 mm, obovoid, ochre to dark yellow, testa smooth.

#### Phenology.

Flowering from June to October; fruiting from September to November.

#### Etymology.

The specific epithet refers to its distribution, Motuo County, Xizang, in China.

#### Vernacular name.

Chinese: 墨脱胡椒 (mò tuō hú jiāo). ‘Mò Tuō’ is a place name, which is the literal translation of the specific epithet *motuoense* and ‘hú jiāo’ is the Chinese name of *Piper*.

#### Habitat and distribution.

**Figure 3.**
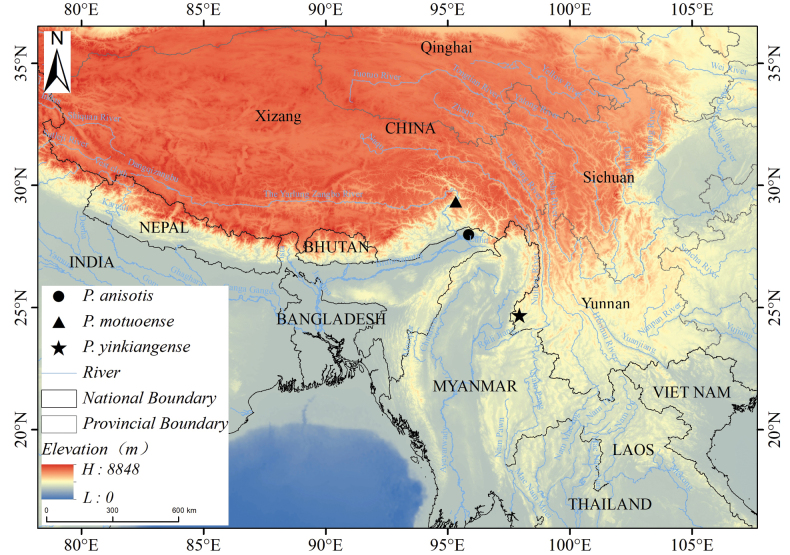
The geographical distribution of *P.motuoense* sp. nov., *P.yinkiangense* and *P.anisotis*.

The new species is currently known only from its type locality in Beibeng Town, Motuo County, Xizang Autonomous Region (Fig. 3). The new species grows very well in the type locality, as it has been recorded in three different sites (Beibeng, Damu and Tiger’s Mouth). It occurs in wet tropical rainforest at elevations of 490–1700 m and often climbs on taller trees or rocks.

#### Comments.

*P.motuoense* was initially misidentified as *P.anisotis* from India, and Motuo is close to Assam in India. After examination of material of *P.anisotis* we found that the new species differs in a number of characters. We also compared it with *P.yinkiangense*. The differences amongst these three species are summarised in Table 1.

## Supplementary Material

XML Treatment for Piper motuoense

## ﻿Additional information

### Author ORCIDs

Fan Su https://orcid.org/0000-0001-7223-6133

Xiao-Wei Qin https://orcid.org/0000-0002-4930-0632

Xun-Zhi Ji https://orcid.org/0000-0003-1835-9145

Chao-Yun Hao https://orcid.org/0000-0002-7541-852X

### Data availability

All of the data that support the findings of this study are available in the main text.
